# The next frontier in lung development and regeneration research: harnessing iPSC models to illuminate notch signaling pathways

**DOI:** 10.3389/fcell.2025.1672074

**Published:** 2025-09-15

**Authors:** Yuetong Song, Sha Yang, Timothy Lam, Henry T. Quach, Jielin Yang, Rasha Salih, Amy P. Wong

**Affiliations:** ^1^ Program in Developmental and Stem Cell Biology, The Hospital for Sick Children, Toronto, ON, Canada; ^2^ Department of Laboratory Medicine and Pathobiology, University of Toronto, Toronto, ON, Canada

**Keywords:** notch signaling, lung development, airway epithelial cells, lung differentiation, induced pluripotent stem cells, regeneration and repair

## Abstract

The respiratory system relies on a diverse repertoire of epithelial cell types to ensure efficient air conduction and gas exchange. This cellular heterogeneity arises through tightly coordinated intercellular signaling events that extend from embryonic development into the postnatal period. Among the key regulatory pathways, Notch signaling plays an integral role in guiding cell fate determination, proliferation, and differentiation. It is indispensable for the proper formation, maintenance, and repair of the airway epithelium. This review examines the broad influence of Notch signaling on mammalian airway epithelial biology and highlights unresolved questions—particularly those specific to human lung development—where human induced pluripotent stem cell–derived models offer promising tools to bridge existing knowledge gaps.

## Introduction

The mammalian lung is the primary site of gas exchange, comprising anatomically and functionally distinct compartments. The upper airway includes the nasal and oral cavities, pharynx, and larynx, whereas the lower airway consists of the trachea, bronchioles, and alveolar sacs. Within the lower airway, proximal regions (trachea and bronchi) function chiefly in air conduction, while distal regions (respiratory bronchioles and alveoli) are specialized for gas exchange, each harboring unique epithelial niches (Weibel, 2017; Van Scott et al., 2013; Pohunek, 2004; Banov, 1989). Here, we review the role of canonical Notch signaling—a conserved cell–cell communication pathway—in airway progenitor specification, differentiation, and regeneration.

### Human and mouse lung morphogenesis

Lung morphogenesis proceeds through five stages in both humans and mice: embryonic, pseudoglandular, canalicular, saccular, and alveolar. The first four stages occur *in utero*; alveologenesis extends postnatally (up to ∼2 years in humans; until postnatal day 28 in mice) (Nikolić et al., 2018; Swarr and Morrisey, 2015; Cardoso and Lü, 2006). During the embryonic phase (mouse E9–E12; human 3–5 gestational weeks [GW]), the NKX2.1+ endodermal buds evaginate from the ventral foregut, forming the trachea and primary lung buds. These buds branch into secondary (three on the right, two on the left) and tertiary bronchi, yielding ten branch tips per lung by stage end (Nikolić et al., 2018; Swarr and Morrisey, 2015; Cardoso and Lü, 2006). In the pseudoglandular stage (mouse E12–15; human 5–16 GW), iterative branching establishes the conducting airway tree; distal tips remain progenitor-rich, while stalk cells begin fate specification (Nikolić et al., 2018; Swarr and Morrisey, 2015; Cardoso and Lü, 2006). The canalicular (mouse E15–17; human 17–24 GW) and saccular stages (mouse E17–postnatal day [P]0; human 24–38 GW) generate respiratory bronchioles and saccules, which mature into alveoli during alveologenesis (mouse P0–20; human 38 GW–2 years) (Nikolić et al., 2018; Swarr and Morrisey, 2015; Cardoso and Lü, 2006).

Progenitor behavior is patterned along the proximal–distal axis, with region-specific niches guiding proliferation, migration, and differentiation (Eenjes et al., 2022; Zhang et al., 2024). In mice, pulmonary neuroendocrine cells (PNECs), expressing CGRP and ASCL1, originate proximally and coalesce into clusters at airway branch points before dispersing into terminal bronchioles (Noguchi et al., 2020; Candeli and Dayton, 2024). They are important during early fetal lung development as signaling hubs, and play roles in oxygen sensing, niche signaling to modulate nearby stem cells, and immune responses (Branchfield et al., 2016; Xu et al., 2020). Proximal–distal patterning underpins adult lung homeostasis and repair through WNT, BMP/TGFβ, and Notch signaling interplays (Hussain et al., 2017; Frum et al., 2023; Rey et al., 2022; Kiyokawa and Morimoto, 2020; Cumplido-Laso et al., 2023).

**FIGURE 1:**
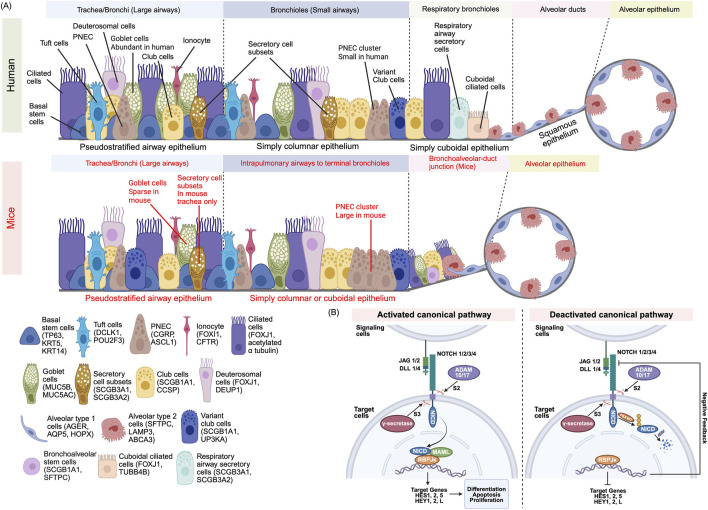
**(A)** Cellular Composition of the Mammalian Airway Epithelium. The mammalian airway epithelium exhibits region-specific cellular diversity. The proximal airway epithelium is composed of multiple specialized cell types, each marked by distinct lineage-defining genes: Basal stem cells (TP63+KRT5+) are resident stem cells that play a role in regenerating the epithelium, Club cells (SCGB1A1+) secrete protective proteins, Goblet cells (MUC5AC+) produce mucus, Deuterosomal cells (DEUP1+) are ciliated cell precursors, Ciliated cells (FOXJ1+) drive mucociliary clearance, Ionocyte (FOXI1+) regulate ion transport, Tuft cells (DCLK1+) function in chemo sensation, and Pulmonary neuroendocrine cells (PNEC) (CGRP+) mediate endocrine signaling. In human respiratory bronchioles, respiratory secretory cells (SCGB3A1^+^) and cuboidal ciliated cells (TUBB4B^+^) protects the airways through mucociliary transport, whereas in mice, the distal airway epithelium is connected by a bronchioalveolar duct junction. The distal airway epithelium is involved in respiratory gas exchange and surfactant production and consists of Alveolar type 1 (AGER+) which facilitates gas exchange and Alveolar type 2 cells (SFTPC+) which secrete surfactants. Mouse-specific markers are highlighted in red, while human’s features are shown in black. **(B)** Canonical Notch Signaling: Activation and Termination. Mammalian cells utilize four Notch receptors (NOTCH1–4) and four canonical ligands (JAG1, JAG2, DLL1, DLL4) to regulate cell fate decisions. In the activation process, ligand binding initiates cleavage at the S2 site via ADAM10/17. A second cleavage at the S3 site by γ-secretase releases the Notch intracellular domain (NICD). The NICD translocates to the nucleus and forms a transcriptional complex with RBPJκ (also known as CSL) and MAML1/2. This complex activates target genes such as HES and HEY, switching RBPJκ from a transcriptional repressor to an activator. During the signal termination process, NICD is tagged for degradation by FBXW7-mediated ubiquitination. Proteasomal degradation of NICD halts signaling. HES/HEY proteins may also provide negative feedback by repressing Notch pathway components.

The proximal pseudostratified epithelium contains basal stem cells (BSC), expressing P63 and KRT5, club cells (SCGB1A1+, CCSP+), secretory cell subset (SCGB3A1+, SCGB3A2+) (Reynolds et al., 2002), goblet cells (MUC5AC+), deuterosomal cells (FOXJ1+, DEUP1+), ciliated cells (FOXJ1+, acetylated α-tubulin+), tuft cells (DCLK1+), and ionocytes (FOXI1+, CFTR+) (Montoro et al., 2018; Plasschaert et al., 2018) (Figure 1A). Basal cells maintain epithelial turnover and mediate repair post-injury (Rock et al., 2009); deuterosomal cells are ciliated progenitor cells (Deprez et al., 2020); ciliated and secretory cells drive mucociliary clearance and barrier function (Bustamante-Marin and Cilia, 2017); tuft cells sense luminal stimuli; ionocytes regulate ionic homeostasis and mucus hydration (Yuan et al., 2023). Distally, epithelium transitions to simple columnar and cuboidal bronchiole cells, culminating in squamous alveolar type 1 cells (AT1), expressing AGER and AQP5 and alveolar type 2 cells (AT2), expressing SFTPC and ABCA3 (Weibel, 2017; Van Scott et al., 2013; Banov, 1989).

Notably, humans and mice differ in epithelial distribution. In humans, the pseudostratified epithelium spans the entire human conducting airway from the trachea to the bronchioles but in mice, this is confined to the trachea and main bronchi (Nikolić et al., 2018). Human airways contain abundant goblet cells in the proximal airways and abundant club cells in the distal regions, whereas mouse distal lungs contain sparse goblet but abundant club cells (Danopoulos et al., 2019). The mouse trachea contains a subset of secretory cells, marked by SCGB3A1 and SCGB3A2 expression, which are found near club and goblet cells; however, in human airways, these cells are confined to the trachea and main the bronchi (Reynolds et al., 2002). PNECs form clusters at mouse bronchiolar neuroepithelial bodies but are solitary in humans (Danopoulos et al., 2019). Distally, human terminal bronchiole cells transition to respiratory bronchiole cells including respiratory airway secretory cells (SCGB3A1+, SCGB3A2+), club cells, and cuboidal ciliated cells (FOXJ1+, TUBB4B+) (Basil et al., 2022; Dodd et al., 2024). In mice, the terminal bronchiole cells directly transition to alveolar ducts through a bronchoalveolar duct junction, containing bronchoalveolar stem cells alongside club, ciliated, and alveolar cells (Kim et al., 2005). This cellular distribution reflects species-specific adaptations and has implications for how airway diseases like asthma or COPD manifest and respond to treatment in humans versus mice. Understanding these species-specific differences in airway architecture and cell distribution provides essential context for exploring the molecular mechanisms, such as Notch signaling, that govern epithelial cell fate and differentiation.

### Canonical notch signaling

Mammals express four Notch receptors (NOTCH1–4, single pass transmembrane protein) and five ligands (JAG1/2, DLL1/3/4), four of them are canonical ligands (JAG1/2, DLL1/4) (Gomi et al., 2015; Stupnikov et al., 2019). Canonical activation (Figure 1B) ensues when ligand binding induces ADAM10/17-mediated S2 cleavage at extracellular juxtamembrane site, and γ-secretase–mediated S3 cleavage, liberating the Notch intracellular domain (NICD). NICD translocates to the nucleus, where it associates with RBPJκ also known as CSL and MAML1/2 cofactors, to drive HES/HEY transcriptional programs as the RBPJ κ (CSL) converts from co-repressor to co-activator (Alabi et al., 2018; D’Souza et al., 2010). To terminate the signaling pathway, NICD is ubiquitinated by FBXW7 and degraded in the proteasome while HES/HEY proteins can auto-regulate and repress Notch ligand/receptor expression as a negative feedback loop (Kar et al., 2021). Non-canonical Notch pathways—independent of CSL or γ-secretase—also contribute to context-specific outcomes but are less well characterized in the lung (D’Souza et al., 2010).

### Notch signaling in mouse airway development

Notch pathway components are expressed from the earliest lung bud stages (mouse E9–12), orchestrating proximal–distal fate decisions (Tsao et al., 2008; Kong et al., 2004). Pharmacological γ-secretase inhibition (DAPT) in E8.5 CD1 mouse embryos foregut explants expand distal *Nkx2.1+* tip progenitors while depleting *Sox2+* proximal progenitors; similarly, DAPT during branching morphogenesis yields enlarged distal buds, dysregulated distal markers (*Nkx2.1, Bmp4, Sftpc*), and reduced *Sox2* (Tsao et al., 2008). Pan-epithelial deletion of Pofut1 (Pofut1^F/–;Shh^Cre^/+^)**,** an enzyme required for Notch activation, nearly abolishes *Sox2* expression in E18.5 lungs, underscoring Notch’s necessity for proximal lineage initiation (Tsao et al., 2009).

Spatial and temporal expression patterns of individual Notch receptors and ligands hint at specialized roles. NOTCH1 is enriched in the distal endoderm from E11.5–E13.5, whereas NOTCH2/3 localizes to mesenchyme, as shown by *in situ* hybridization (Post et al., 2000); NOTCH4 is restricted to vasculature (Ito et al., 2000; Uyttendaele et al., 1996). *Notch1/2/3* genes are expressed from E11– P14, with *Notch1* detectable as early as E10 and continuing postnatally, based on semi-quantitative RT-PCR (Kong et al., 2004). Notch ligands also exhibit dynamic expression patterns. *Jag1* and *Jag2* are dynamically expressed from E11–P14, with *Jag2* peaking at E16.0 and preceding *Jag1* onset (Stupnikov et al., 2019; Kong et al., 2004; Zhang et al., 2013); *Dll1* emerges in secondary bronchi at E13.5 and intensifies in bronchioles and branch points until birth (Post et al., 2000). These patterns align with Notch’s central role in branching morphogenesis and regionalization.

**FIGURE 2:**
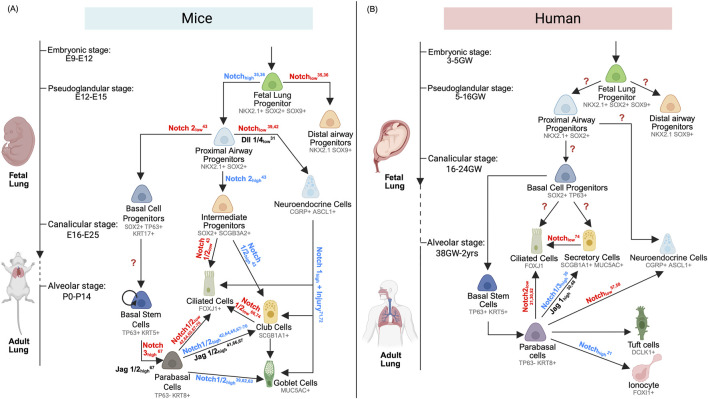
**(A)** Mouse Proximal Airway Development and Regeneration. Dynamic Notch signaling orchestrates cell fate specification throughout mouse airway development and repair. From embryonic stages (E9–12) to alveolar maturation (P0–14), Notch activity directs NKX2.1+SOX2+SOX9+ fetal lung progenitors toward either distal (NKX2.1+SOX9+) or proximal (NKX2.1+SOX2+) lineages. Proximal progenitors give rise to basal cell progenitors (SOX2+TP63+), pulmonary neuroendocrine cells (CGRP+), and intermediate progenitors (SOX2+SCGB3A2+). Basal cell progenitors mature into basal stem cells (TP63+KRT5+), which, during development or regeneration, generate parabasal cells (TP63-KRT8+) that differentiate into ciliated (FOXJ1+), club (SCGB1A1+), and goblet (MUC5AC+) cells. Intermediate progenitors also contribute to ciliated and club cell lineages during development. During regeneration, PNEC can give rise to all three secretory and ciliated cell types. Notch inhibition enables club cells to transdifferentiate into ciliated cells. **(B)** Human Proximal Airway Development and Regeneration. In humans, Notch signaling similarly governs airway lineage specification from embryonic (3–5 gestational weeks) to alveolar stages (38 GW–2 years). NKX2.1+SOX2+SOX9+ fetal lung progenitors are directed toward distal (NKX2.1+SOX9+) or proximal (NKX2.1+SOX2+) fates. Proximal progenitors differentiate into basal cell progenitors (SOX2+TP63+) and pulmonary neuroendocrine cells (CGRP+). During fetal development, basal progenitors can directly form ciliated (FOXJ1+) and secretory cells (SCGB1A1+, MUC5AC+) or mature into basal stem cells (TP63+KRT5+) that regenerate the airway epithelium via a parabasal intermediate. In regeneration, Notch signaling also promotes differentiation of ionocytes (FOXI1+) and PNEC (CGRP+). Goblet cells (MUC5AC+) can transdifferentiate into ciliated cells upon Notch inhibition.

Within the proximal epithelium, Notch directs the emergence of cellular diversity (Figure 2A). Loss of Notch signaling—via receptor knockouts or downstream effectors ablation (HES1)—enhances NE bodies (NEB) size and PNEC differentiation (Ito et al., 2000; Morimoto et al., 2012), with DLL1/4 acting as dominant ligands; *Dll1/Dll4* double knockouts show marked increases in *Ascl1*+ progenitors that co-express *Cgrp* upon PNEC maturation (Stupnikov et al., 2019). Post-PNEC specification, *Notch2* activation in *Sox2+* proximal airway progenitors fosters *Scgb3a2+* intermediate progenitors (Kiyokawa et al., 2021), which adopt secretory or ciliated fates depending on subsequent *Notch2* signaling levels (Kiyokawa et al., 2021). *Notch1*/*2* alleles dose-dependently regulate secretory cell differentiation (Morimoto et al., 2012; Guseh et al., 2009; Boucherat et al., 2012), with *Notch2* playing a dominant role in generating *Cc10+* secretory cells (Morimoto et al., 2012). In contrast, *Sox2+* progenitors lacking *Notch2* activation differentiate into *Krt17* ^*+*^ basal progenitors (Kiyokawa et al., 2021). Collectively, these studies suggest an important temporal and possibly spatial role of Notch signaling in regulating cell fate during airway morphogenesis.

### Limited studies on NOTCH regulation during human fetal airway development

Although limited, current studies on Notch regulation during human fetal airway development highlight a significant knowledge gap. Most of our understanding of Notch receptor–ligand interactions during fetal airway development derives from murine models, which have elucidated roles for Notch1–3 and ligands Dll1/4 and Jag1/2 in proximal–distal patterning and epithelial differentiation (Stupnikov et al., 2019; Tsao et al., 2008; Tsao et al., 2009; Ito et al., 2000; Zhang et al., 2013; Morimoto et al., 2012; Kiyokawa et al., 2021; Guseh et al., 2009; Boucherat et al., 2012) (Figure 2). In contrast, direct investigation of Notch‐dependent lineage specification in the human fetal lung remains virtually unexplored. Using human embryonic stem cell (hESC) differentiation models, Chen et al. demonstrated that Notch pathway inhibition via DAPT enhances the differentiation of PNECs (Chen et al., 2019). However, to date, human studies have largely been confined to postnatal primary airway cultures, where Notch modulation alters basal, secretory, ciliated, and neuroendocrine cell proportions (Rey et al., 2022; Gomi et al., 2015; Giuranno et al., 2020; Gomi et al., 2016; Kang et al., 2011; Vladar et al., 2023; Bodas et al., 2021; Danahay et al., 2015; Marcet et al., 2011; Bou Saab et al., 2016; Paul et al., 2014). Restricted access to human fetal tissue across developmental stages, coupled with limitations of conventional *in vitro* airway epithelial models, has impeded dissection of ligand–receptor dynamics *in utero*.

Human induced pluripotent stem cell (hiPSC)–based platforms now offer a scalable, stage‐specific system for modeling airway morphogenesis. Directed differentiation of hiPSCs recapitulates successive milestones from definitive endoderm to mature, pseudostratified airway epithelium, enabling temporal control of Notch pathway perturbation (Wang et al., 2023). Two independent hiPSC studies demonstrate that γ‐secretase inhibitors (DAPT or DBZ) expand BSC, ciliated, and pulmonary neuroendocrine cell (PNEC) populations while suppressing club cell differentiation—phenocopying murine embryonic lung responses to Notch blockade (de Carvalho et al., 2019; Hor et al., 2020). Although promising, these initial reports rely on pan‐Notch inhibition; future work must leverage ligand‐ and receptor‐specific manipulations, coupled with single‐cell and organoid assays, to resolve how distinct Notch inputs sculpt human airway lineage hierarchies.

Overall, hiPSC‐derived airways, human lung organoids, and *ex vivo* fetal explants represent complementary systems for untangling the multifaceted roles of Notch signaling in human lung development. Strategic application of these models will be essential to define the temporal and spatial rules by which Notch ligand–receptor codes drive epithelial patterning in the human fetus.

### Notch signaling in mouse airway regeneration

The adult airway epithelium undergoes continual turnover—estimated to be every 30–50 days in rodents and there are limited studies in humans (Bowden, 1983)—and relies on BSCs for homeostatic replenishment and repair after injury (Rock et al., 2009; Travaglini et al., 2020; Mou et al., 2021; Wu et al., 2022). In both mouse and human adult airways, BSCs act as resident stem cells capable of self-renewal and regenerating various proximal airway epithelial cell types. Notch signaling plays a critical role in maintaining mature airway epithelial cells homeostasis and promoting injury-induced repair by orchestrating BSC differentiation, directly progenitors toward secretory or ciliated lineages (Eenjes et al., 2022; Wu et al., 2022; Sriu et al., 2002) (Figures 2A,B).

In mouse conducting airway, *Notch1/2* activation is both necessary and sufficient to promote club and goblet cell differentiation (Morimoto et al., 2012; Morimoto et al., 2010; Lingamallu et al., 2024). Conversely, inhibition of *Notch1*/*2* promotes ciliated cell differentiation in adult mouse airway epithelial cells grown in air-liquid interface (ALI) cultures (Lafkas et al., 2015). JAG1/2 also plays a critical role in balancing distinct cell populations in adult mice conducting airways (Zhang et al., 2013; Lafkas et al., 2015; Mori et al., 2015) (Figure 2A). Ligands JAG1/2 maintain the secretory compartment—neutralizing JAG1 or JAG2 selectively using antibodies depletes club cells while expanding ciliated cells in adult mice airways with a more prominent effect observed with JAG1 inhibition than JAG2, and combined JAG1/2 blockade yields an almost exclusively ciliated epithelium (Lafkas et al., 2015). Therefore, JAG1/2-activated Notch signaling is crucial for the development of secretory cells, while its inhibition favors ciliated cell development, as confirmed in several studies (Guseh et al., 2009; Boucherat et al., 2012; Morimoto et al., 2010; Ou-Yang et al., 2013) (Figure 2A). *Notch3*, by contrast, activated by JAG1/2 drives *Krt8+, Tp63–* parabasal progenitor formation (Mori et al., 2015), a distinct intermediate state that later activates Notch1/2 for secretory and ciliated cell fate decision (Mori et al., 2015; Rock et al., 2011; Pardo-Saganta et al., 2015). Single‐cell RNA sequencing in adult mouse airways further implicates Notch in the specification of rare epithelial types: *Ascl1* in PNECs, *Ascl2* in tuft cells, and *Ascl3* in ionocytes, and identifies *Nfia* as a club cell–enriched modulator of Notch‐dependent homeostasis (Noguchi et al., 2020; Montoro et al., 2018).

Following chemical injuries such as naphthalene, sulfur dioxide, chlorine gas, or polidocanol treatment, Notch signaling is a key pathway for efficient regeneration in mouse airways (Paul et al., 2014; Morimoto et al., 2010; Rock et al., 2011; Pardo-Saganta et al., 2015; Ouadah et al., 2019; Yao et al., 2018; Xing et al., 2012). In naphthalene models, absence of Notch signaling in PNECs impairs their conversion into club, ciliated, and goblet cells (Ouadah et al., 2019; Yao et al., 2018), while Notch inhibition in goblet cells accelerates ciliated transdifferentiation (Ruiz Ga et al., 2019). Sulfur dioxide and chlorine injuries activate Notch + parabasal cells (*Tp63–, Krt8+)* to replenish luminal progenitors (Rock et al., 2011; Pardo-Saganta et al., 2015). Within BSCs, NICD2 marks cells fated for secretory lineages, whereas *c-myb*‐expressing BSCs yield ciliated progeny; *Rbpjκ* deletion shifts the balance toward ciliated cell lineages (Pardo-Saganta et al., 2015). In polidocanol‐injured airways, elevated reactive oxygen species engage NRF2, a key transcription factor that regulates oxidative stress and inflammation via the NRF2-antioxidant response element pathway, to activate NOTCH1 in Notch pathway, driving BSC proliferation and self‐renewal (Paul et al., 2014). Overall, Notch signalling is important in the regeneration of the airways post-injury in mice.

### Notch signaling in human airway regeneration

In human primary ALI and 3D bronchosphere models, broad inhibition of NOTCH signaling using small molecules like DAPT or DBZ reduces the number of secretory cells and ionocytes, while promoting expansion of basal and ciliated cell populations (Rey et al., 2022; Plasschaert et al., 2018; Gomi et al., 2015; Gomi et al., 2016; Danahay et al., 2015; Paul et al., 2014). Conversely, constitutive activation of NOTCH1 and NOTCH3, achieved through lentiviral overexpression of their active intracellular domains (NICD1/3), in human primary BSCs cultured in ALI conditions increases secretory cells (e.g., MUC5AC, SCGB1A1) at the expense of basal (KRT5, TP63) and ciliated (DNAI1, TEKT1) cells (Gomi et al., 2015). These findings highlight the role of NOTCH1/3 receptor activation in modulating differentiation among secretory, basal, and ciliated cell types.

Further evidence from a doxycycline-inducible NICD1 system in iPSC-derived BSC-like cells showed that NOTCH1 activation does not promote ionocyte differentiation (Wang et al., 2023). In contrast, using neutralizing antibodies against NOTCH1 in a 3D human BSC-derived bronchosphere model led to increased expression of BSC markers, without affecting goblet or ciliated cell differentiation (Danahay et al., 2015). Inhibition of NOTCH3 slightly elevated markers associated with both BSCs and goblet cells, but did not alter ciliated cell differentiation (Danahay et al., 2015). Meanwhile, neutralizing NOTCH2 reduced secretory cell populations and increased basal and ciliated cell markers (Danahay et al., 2015).

Additional support for Notch signaling’s role in human airway cell development comes from studies that manipulate NOTCH ligands or downstream transcriptional regulators. For instance, lentiviral overexpression or knockdown of the ligand JAG1 selectively influences the expansion of secretory cell lineages, while leaving ciliated cell populations unaffected (Gomi et al., 2015; Gomi et al., 2016). Furthermore, knockdown of DLL1/NOTCH1 via miR-449 microRNA in deuterosomal cells leads to increased expression of HES6—a Notch pathway inhibitor—and decreased levels of HEY1 and HES4, which are Notch activators, along with reduced expression of Notch receptors 1, 2, and 3. This shift suppresses secretory cell differentiation and enhances multiciliated cell formation in human airway epithelial ALI cultures (Marcet et al., 2011; Ruiz Ga et al., 2019).

Altogether, these findings emphasize the intricate specificity of the NOTCH pathway in directing cell lineage outcomes, which is essential for refining differentiation protocols aimed at generating specialized cell types from human iPSCs.

Disease‐associated analyses implicate aberrant Notch activity in chronic airway disorders. In cystic fibrosis (CF), increased basal proliferation and ciliated cell loss coincides with heightened Notch signaling; DBZ/DAPT treatment restores ciliated abundance in CF epithelium (Vladar et al., 2023; Bou Saab et al., 2016; Barbry et al., 2020). Airway epithelial cells from patients with smoking‐induced chronic obstructive pulmonary disease (COPD) display elevated secretory and reduced basal cell proportions when compared to non-smokers and downregulated expression of Notch pathway genes compared to non-smokers (Tilley et al., 2009). This data highlight Notch pathway modulation as a promising avenue for regenerative therapies aimed at reestablishing balanced epithelial architecture.

### Discrepant outcomes in notch–mediated regulation in airway lineages

While most murine studies consistently show that Notch activation inhibits ciliated cell differentiation and that its suppression promotes multiciliogenesis (Stupnikov et al., 2019; Tsao et al., 2008; Tsao et al., 2009; Morimoto et al., 2012; Guseh et al., 2009; Vladar et al., 2023; Danahay et al., 2015; Lafkas et al., 2015), a few investigations challenge this paradigm. In embryonic mouse lungs, Kiyokawa et al. observed a slight increase in ciliated cells following Notch stimulation, indicating that multiciliogenesis may be influenced by receptor dosage or the timing of activation (Kiyokawa et al., 2021). Similar inconsistencies appear in adult lung injury models. For example, in naphthalene-treated mice, Ouadah and colleagues reported that NOTCH1 activation in NICD1-overexpressing mice suppressed PNEC differentiation (Ouadah et al., 2019), and other studies have shown PNEC expansion following Notch1 knockout mice (Xing et al., 2012). Contrastingly, Morimoto’s group found no significant change in PNEC numbers after manipulating NOTCH 1 signaling (Morimoto et al., 2012).

Human airway studies also reveal conflicting data. In primary human cell cultures, most reports show that Notch inhibition promotes ciliated cell formation (Plasschaert et al., 2018; Giuranno et al., 2020; Vladar et al., 2023; Danahay et al., 2015; Marcet et al., 2011; Bou Saab et al., 2016; de Carvalho et al., 2019), yet others describe decreased multiciliogenesis under similar conditions or after HEYL knockdown (Gomi et al., 2016; Bodas et al., 2021; Hor et al., 2020). Targeted NOTCH3 activation likewise yields mixed outcomes: Gomi et al. documented reduced ciliated markers in NICD3-overexpressing epithelia (Gomi et al., 2015), but Bodas et al. observed no alteration in ciliated cell numbers despite robust NICD3 induction (Bodas et al., 2021).

NOTCH1/3 activation and NOTCH2 inhibition are key in human mature airway cell fate decisions (Gomi et al., 2015; Bodas et al., 2021; Danahay et al., 2015; Hor et al., 2020; Ou-Yang et al., 2013). In mice, NOTCH1/2 is crucial for mature airway cell differentiation, but NOTCH3 is not involved in either activation or inhibition (Morimoto et al., 2012; Kiyokawa et al., 2021; Danahay et al., 2015; Morimoto et al., 2010; Lafkas et al., 2015; Mori et al., 2015). These findings suggest discrepancies between human and mouse Notch signaling activation and inhibition.

Together, these inconsistencies underscore the need to dissect receptor- and ligand-specific contributions, as well as the influence of developmental stage, injury context, and species differences, on Notch-driven airway lineage allocation.

### Harnessing hiPSC-Based models and cutting-edge genomic technologies to elucidate notch signaling dynamics

Many hiPSC-derived airway epithelium differentiation protocols have leveraged Notch signaling regulation, highlighting its importance and potential on lineage regulation during human lung development and homeostasis. Particularly, DAPT had been applied to induce the differentiation of CFTR-expressing ciliated cells in fetal airway organoids (Wong et al., 2015; Ngan et al., 2022; Konishi et al., 2016) and in ALI cultures of mature airway epithelium (Firth et al., 2014). Notch inhibition via DAPT/DBZ has also been shown to induce the differentiation of BSCs, ciliated cells, and PNECs, while reducing club cells differentiation (de Carvalho et al., 2019; Hor et al., 2020). Therefore, the role of Notch signaling in the pulmonary disease—such as lung cancer, CF, and COPD—as well as in airway epithelial repair/regeneration, can be further investigated using hiPSC-derived fetal and adult airway epithelium (Wong et al., 2015; Konishi et al., 2016; Firth et al., 2014; Ahmed et al., 2022; Marcoux et al., 2023).

Human iPSC-derived models of airway epithelium, when integrated with powerful gene-editing platforms such as CRISPR/Cas9 and prime editing, enable the creation of epithelial cell lines with targeted knock-in or knock-out modifications to Notch receptors and transcription factors. These cell lines facilitate receptor-specific investigations across various stages of lung development (Huang and Loewer, 2022). Additionally, cellular barcoding, a technique that reverse-transcriptionally labels individual cells with unique molecular identifiers via lentiviral delivery (Shakiba et al., 2019), can be coupled with high resolution single-cell RNA sequencing technologies to trace airway epithelial lineages and uncover patterns of cellular competition modulated by Notch signaling throughout human lung morphogenesis, using hiPSC-derived cells at defined developmental intervals. These advanced *in-vitro* platforms offer a powerful and versatile system for dissecting Notch-driven mechanisms of airway development, mapping cellular trajectories, and benchmarking engineered cell states against native human lung tissue (Quach et al., 2024).

Despite advances, studies exploring the dynamic interactions between specific Notch receptor-ligand pairs during lung development remain limited. Emerging single-cell spatial transcriptomic platforms (Garcia-Alonso et al., 2021), offer promising avenues for mapping Notch signaling gene expression within hiPSC-derived lung tissues. These technologies may illuminate how Notch signaling influences the spatial organization and fate of epithelial cell lineages in discrete regions of the developing lung, and support inferences regarding canonical Notch-mediated cell-cell communication (Armingol et al., 2022).

## Discussion

In this review, we have highlighted the complex role of Notch signaling in lung development and regeneration. There are extensive studies in mouse models demonstrating the importance of the Notch signaling pathway in development and regeneration, and particularly its role in cellular differentiation. Understandably, there is less research on the role of this pathway in human airways, especially in the context of lung development and disease.

Two studies highlight the essential role of Notch signaling in regulating epithelial basal cell function using hiPSCs and mouse genetic models (Huang et al., 2024; Zhang et al., 2018). These investigations were conducted in the esophagus, an organ that is anatomically and developmentally contiguous with the ventral lung, underscoring their relevance to airway biology and support the broader context of Notch signaling in epithelial maintenance.

Despite the physiological parallels between mice and humans, murine models fall short in fully replicating the entirety of human physiology. In addition, the effects of Notch signaling in early human lung development remain ambiguous. With advancements in human iPSC-derived models combined with effective gene targeting tools and powerful platforms such as cellular barcoding and single cell spatial transcriptomics technologies, these will become important tools to uncover the role of Notch signaling in human lung models. The experimentally tractable models of iPSC differentiation enable temporal studies of the effects of Notch (and other pathways) in lung development and repair.

Overall, future studies leveraging human iPSC-derived airway epithelium models may reveal the complex dynamic role of Notch signaling across development from fetal to “adulthood” (maturation), informing how cells emerge to how they are regenerated and the impact on disease.

## Glossary

AT1: Alveolar type 1
AT2: Alveolar type 2
ASCL1/2/3: Achaete-scute homolog 1/2/3
BSCs: Basal stem cells
BADJ: Bronchoalveolar duct junctions
CSL: CBF1/Su(H)/Lag-1
CALCA: Calcitonin related polypeptide alpha
CFTR: Cystic fibrosis transmembrane conductance regulator
c-myb: C-MYB proto-oncogene
CRISPR: Clustered Regularly Interspaced Short Palindromic Repeats
DLL1/3/4: Delta-like 1/3/4
E: Embryonic day
FOXI1: Forkhead box I1
FOXJ1: Forkhead. box J1
GW: Gestational week
DAPT/DBZ: γ-secreatase inhibitor
HAEC: Human airway epithelial cells
hiPSC: Human induced pluripotent stem cell
ISH: *In situ* hybridization
JAG1/2: Jagged1/2
KRT5: Keratin five
KRT8: Keratin8
Lfng: Lunatic Fringe proteins
MAML: Mastermind-like proteins
MUC5ac/5b: Mucin 5ac/5b
NE: Neuroendocrine
NICD: Notch intracellular domain
NEB: NE bodies
NFIA: Nuclear factor IA
NRF2: Nuclear factor erythroid-2-related factor 2
PNEC: Pulmonary neuroendocrine cells
RBPJ*κ*: Recombination Signal Binding Protein For Immunoglobulin Kappa J Region *κ*
Rfng: Radical Fringe proteins
**SO**_**2**_: Sulfur dioxide
P63: Tumor protein 63 (Protein)
Trp63: Transformation related protein 63
